# Synthesis of a cell penetrating peptide modified superparamagnetic iron oxide and MRI detection of bladder cancer

**DOI:** 10.18632/oncotarget.13578

**Published:** 2016-11-24

**Authors:** Chen Ding, Kaijie Wu, Weiyi Wang, Zhenfeng Guan, Lei wang, Xinyang Wang, Rong Wang, Li Liu, Jinhai Fan

**Affiliations:** ^1^ Department of Urology, The First Affiliated Hospital of Xi’an Jiaotong University, Xi’an, China; ^2^ Department of Urology, Xiangyang Central Hospital, Hubei University of Arts and Science, Hubei Province, China; ^3^ Department of Thoracic Surgery, Tangdu Hospital, The Forth Military Medical University, Xi’an, China; ^4^ Oncology Research Lab, Key Laboratory of Environment and Genes Related to Diseases, Ministry of Education, Xi’an, China; ^5^ Department of Radiology, The First Affiliated Hospital of Xi’an Jiaotong University, Xi’an, China; ^6^ Department of Radiology, The University of Texas Southwestern Medical Center, Dallas, TX, USA

**Keywords:** bladder cancer, superparamagnetic iron oxide, cell penetrating peptide, magnetic resonance imaging, targeted diagnosis

## Abstract

Bladder cancer is the most common malignancy of the urinary tract for which the accurate measurement of minimal residual disease is critical to treatment and determining prognosis. Although cystoscope examination and voided urine cytology remain the current standard of care for detecting residual disease, these approaches are limited by mechanical trauma and lack sensitivity. To develop a new accurate noninvasive method, we developed a novel contrast agent where the surface of superparamagnetic iron oxide (SPIO) nanoparticles is functionalized with a bladder cancer-specific fluorescein isothiocyanate (FITC) labeled cell penetrating peptide (CPP)-polyarginine peptides (R11) for active targeting and imaging. The stable nanoparticles have an average hydrodynamic diameter of 51 nm, surface charge of -21 mV and MRI *r_2_* relaxivity 135 mM^−1^s^−1^. *In vitro* cell studies demonstrated that the R11-conjugated SPIO (SPIO-R11) nanoparticles were taken up by bladder cancer cells (T24) in a dose-dependent manner, which was higher than unconjugated SPIO. TEM showed that SPIO-R11 was mainly concentrated on cell vesicle and lysosome, not in cell nucleus, and no obvious damage was seen on cell ultrastructure. Moreover, uptake of the nanoparticles showed significantly more SPIO-R11 accumulation in bladder cancer cells than in immortalized bladder epithelial cells unlike control SPIO. Further, SPIO-R11 was compatible with immortalized bladder epithelial cells at all tested concentrations up to 200 μg/mL after 72 h incubation. Moreover, SPIO-R11 decreased the magnetic resonance *T_2_* relaxation time by 73% in tumors cells *in vitro* compared to 12% with SPIO. These results indicate great potential of SPIO-R11 as contrast agent to target bladder cancer for diagnostic and therapeutic applications.

## INTRODUCTION

Bladder urothelial cell carcinoma (UCC), the 9^th^ most common cancer diagnosis worldwide [1], is the most common malignancy of the urinary tract. Progression of stage and/or grade occurs in 10% to 50% of cases. Consequently, rigorous surveillance is advocated. Cystoscopy and cytology are the standard methods used to detect and monitor UCC [2]. Cystoscopy has a high sensitivity, but it is invasive difficult to detect the flat malignancies such as Tis [3]. Cytology has the advantage of being noninvasive with a high specificity, but it lacks sensitivity, especially for low grade disease [4]. Magnetic resonance imaging (MRI) is one of the most powerful noninvasive techniques today in clinical medicine for tumor diagnosis. Due to its capability for revealing three dimensional anatomic details without any damage and providing high spatial resolution non-invasively, it attracts intense interest in clinical studies. However, MRI is less sensitive than nuclear medicine or fluorescence imaging when used to monitor small tissue lesions, cellular activities or molecular activity [5, 6]. Therefore, development of new contrast agents is expected to enhance the precision of MRI.

There are various types of contrast agent on the market. The most widely used contrast agents in MRI are the paramagnetic gadolinium (Gd^3+^) complex-based contrast agents and superparamagnetic iron oxide (SPIO) nanoparticles. Compared with the former, SPIO are good alternatives as contrast agents for MRI, because they can generate intense MR signal contrast several orders of magnitude higher than traditional gadolinium based contrast agents [9, 10]. Moreover, they have low toxicity are biocompatibile and exhibit superparamagnetic properties [11–13]. Important parameters for SPIO to be used as contrast agents are their outstanding magnetic properties and surface coating properties as a versatile platform for conjugation of a variety of moieties for cell delivery and targeting [14, 15]. With the help of SPIO, MRI has made great progress in studying gene and drug delivery, cell trafficking, tumor diagnosis, cancer-targeted therapy and many other fields [8, 16–19]. Among these SPIO contrast agents, dextran- or carboxydextran-coated SPIO nanoparticles are the most commonly used and clinically approved such as Feridex or Resovist [20].

Previously, a study of human urinary bladder tumors showed that a tumor as small as 4 mm could be detected using SPIO with a 1.5 T whole-body MR imager, but it was not possible to reliably differentiate the layers of the bladder wall [21]. The main reason is that the plain SPIO are not readily internalized by bladder tumor cells without the use of transfection reagents. Typically, less than 1% of SPIO is internalized by cellular nonspecific endocytosis [22, 23]. This low level of incorporation stresses the already low sensitivity of MRI technology. Accordingly, researchers have conjugated a variety of agents to nanoparticles, including SPIO, to increase the percentage uptake of nanoparticles [24, 25]. Cell penetrating peptide (CPP) is supposed to be one of the best transport vector tools to improve active internalisation of nanoparticles into a target cell [26]. It has the ability to transport diverse types of cargo molecule efficiently across cell membrane. In addition, functional groups can be introduced at the desired site of CPP easily to achieve site-specific conjugation, and their small size relative to aptamers and antibodies makes it possible to attain a high degree of conjugation [27–29]. For example, Tat peptide and RGD conjugated iron-oxide based nanoparticles were used for intracellular magnetic labeling or targeting of tumor angiogenesis by MRI [30–34]. Yet one of the major limitations of these approaches is that the cellular uptake of the majority of CPPs is not cell-type- and organ-specific, so that screening a variety of CPPs for optimal intracellular delivery into human urothelial cell lines is imperative. Our previous study showed that the poly(11)-arginine, termed R11, compared with some other CPPs, exhibited the highest uptake efficiency by different bladder cancer cell lines [35]. Similarly, *in vivo* evaluation of the tissue distribution of R11 in nude mice showed that R11 exhibited an organ-specific uptake in bladder and prostate tissues after intravenously delivery [36]. These results suggest that R11 could be used as a potential delivery vehicle for bladder tumor-targeted diagnosis and therapies.

In the present study, we describe the development of a bladder-specific-peptides-conjugated SPIO (SPIO-R11) for use in targeted cancer imaging with MRI, investigating its synthesis and characterization, cytotoxicity, ability to enter bladder cancer cells *in vitro*, and the effectiveness as a MR contrast agent.

## RESULT

### Characterization of SPIO-R11

**Figure 1:**
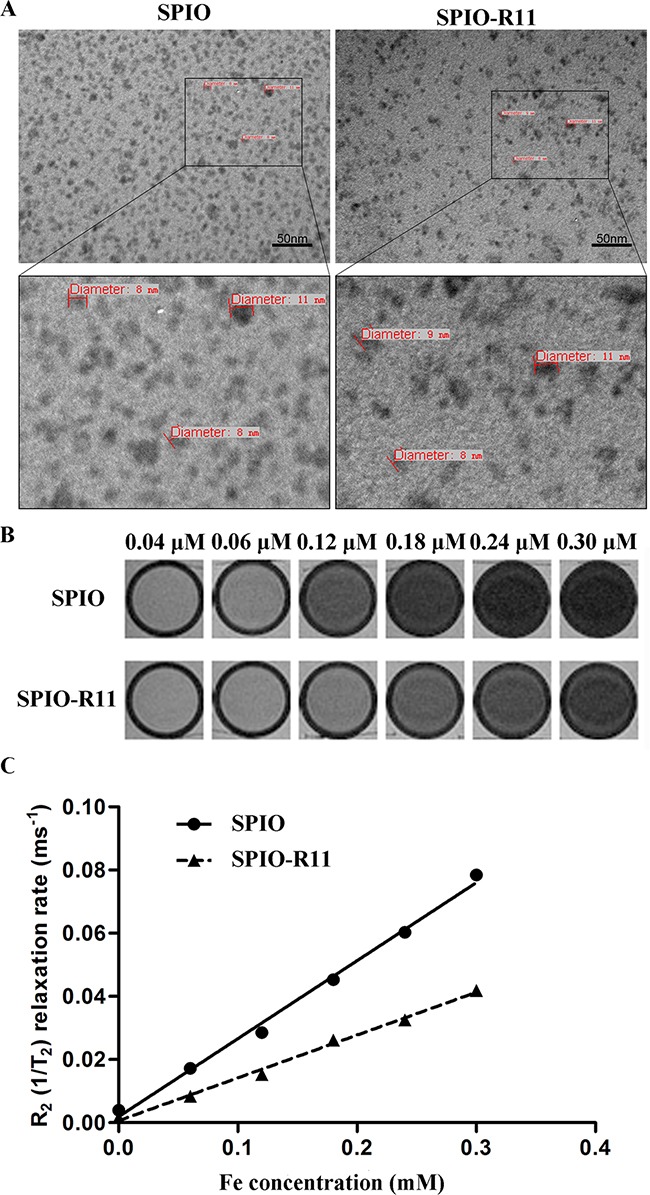
A. TEM image of SPIO and SPIO-R11 Top row represents nanoparticles at a magnification of 200,000×. The black box outlines the area magnified and presented in the bottom row showing both the nanoparticles without obvious aggregation. The small dark spheres were the iron oxide cores of the nanoparticles and the size of cores is around 8-11 nm. Scale bar, 50 nm. **B**. T_2_-weighted MR images (GE 3.0T, multiple spin echo sequence: TR=4000 ms, TE=60 ms) of the above nanoparticles at different concentrations. **C**. R_2_ relaxation rates (1/T_2_, s^−1^) of SPIO and SPIO-R11 as a function of Fe concentration in water at a magnetic field of 3T at room temperature. The *r_2_* values of SPIO and SPIO-R11 were 232 mM^−1^s^−1^ and 135 mM^−1^s^−1^, respectively.

R11 peptides were conjugated to aminated SPIO via SPDP chemistry for targeting of bladder cancer described in the Materials and Methods section. TEM analysis of the nanoparticle size and morphology was conducted by evaporating water from the dispersion on amorphous carbon coated copper grid. Representative TEM images of SPIO and SPIO-R11 are depicted in Figure 1A. The nanoparticles were evenly distributed with no obvious aggregation. The bottom row of Figure 1A shows that the core size of nanoparticle is around 8-11 nm in diameter and morphology is spherical in shape. In comparison with SPIO, there is no observable change in core size, morphology and distributed after surface modification with R11. *T_2_* relaxation times were measured from regions of interest before and after functionalization and results were inverted to obtain the *R_2_* relaxation rates. As shown in Figure 1B, both SPIO and SPIO-R11 tend to darker with the increasing Fe concentration, indicating that these nanoparticles can effectively shorten the spin-spin relaxation time of water protons in *T_2_*-weighted MR images. The measured *r_2_* values of SPIO and SPIO-R11 were 232 mM^−1^s^−1^ and 135 mM^−1^s^−1^ respectively (Figure 1C). To further examine the colloidal stability of SPIO-R11, we monitored changes in their hydrodynamic diameter, PDI, and surface charge. The results are represented in Table 1. The hydrodynamic diameter of SPIO and SPIO-R11 was 37.97 nm and 51.36 nm respectively, which was larger than dehydrated nanoparticles in TEM. The dynamic light scattering (DLS) results also indicated a very narrow range of polydispersity in SPIO (PDI=0.238) and SPIO-R11 (PDI=0.237). In addition, the results of zeta potentials measurements indicated that SPIO (24.53 mV) and SPIO-R11 (−20.67 mV) both have the high absolute values of surface charge.

**Table 1:** Physicochemical properties of SPIO and SPIO-R11

Sample	Hydrodynamic diameter (dv/nm)	PDI	Zeta potential (mV)
SPIO	37.97	0.238	24.53
SPIO-R11	51.36	0.237	−20.67

### Cellular uptake of SPIO and SPIO-R11

**Figure 2:**
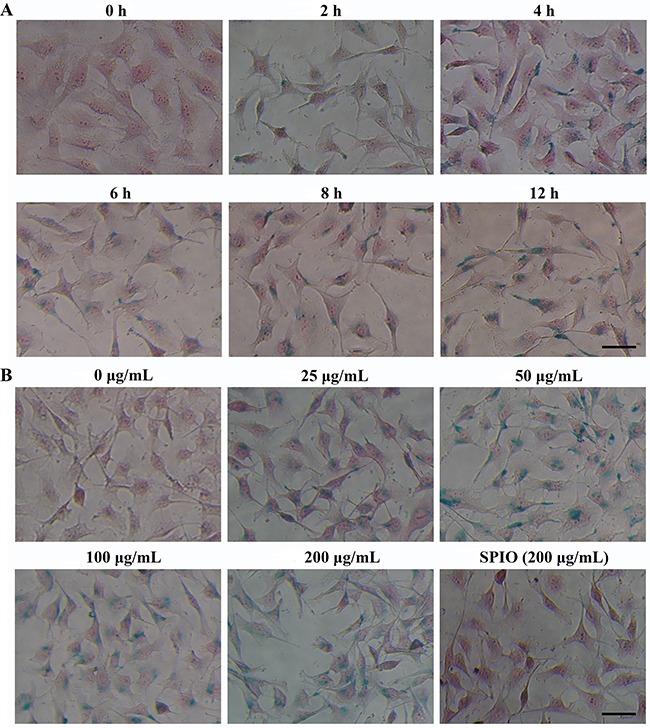
Prussian blue-stained and nucleus fast red-counterstained T24 cells incubated with SPIO-R11 or SPIO **A**. T24 cells were incubated with SPIO-R11 at an iron concentration of 50μg/mL of growth medium for indicated time intervals (0 to 12 h). Nanoparticle uptake increased for longer incubation times, especially at the time range of 0-4 h. **B**. T24 cells were incubated with various iron concentrations of the SPIO-R11 (0 μg/mL, 25 μg/mL, 50 μg/mL, 100 μg/mL and 200 μg/mL) and SPIO (200 μg/mL) for 4 h. A dose-dependent and higher uptake of SPIO-R11 (blue granules) compared with SPIO clearly indicated. The bar in the bottom right corner represents 50 μm.

To first determine the optimal time for incubation, T24 cells were incubated with SPIO-R11 for different time intervals. The uptake of SPIO-R11 was then assessed histologically using Prussian blue staining (Figure 2A). The results showed that there was a time-dependent increase in uptake of SPIO-R11 in the time range of 0-4 h. After more than 4 h incubation, however, the differences in uptake of SPIO-R11 were less prominent. Then cellular uptake of the SPIO-R11 and SPIO by two different cells (T24 and SV-HUC) was performed to determine the R11-mediated cellular uptake efficiency and the optimal concentration of the nanoparticles. As shown in Figure 2B, the cellular uptake efficiency of SPIO-R11 increases with the increase of Fe concentration. Although SPIO-R11 were internalised in both the cell lines, the quantity in SV-HUC cells was much less compared to that in T24 cells (Supplementary Figure S1). Furthermore, SPIO-R11 cellular uptake by T24 cells tend to be saturated at an iron concentration of 50 μg/mL. However, the control studies using SPIO showed very little intracellular uptake of nanoparticles in both the cell lines.

**Figure 3:**
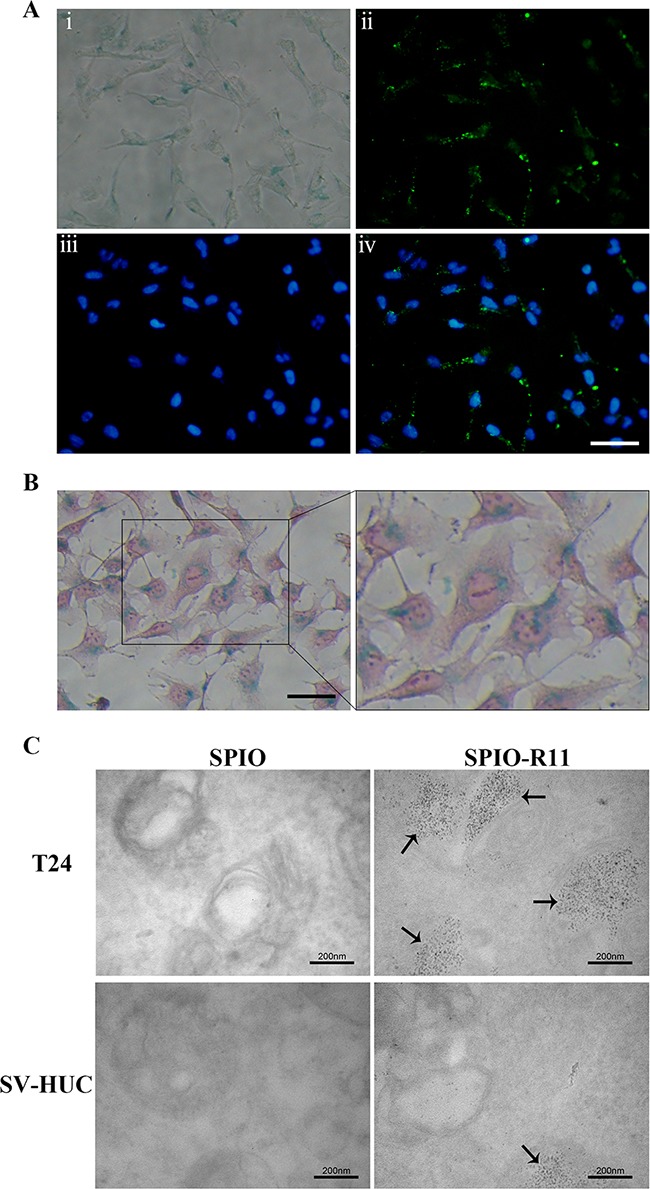
Localization of nanoparticles in T24 cells T24 cells were incubated with nanoparticles at an iron concentration of 50 μg/mL for 4 h. **A**. T24 cells were incubated with SPIO-R11 and then washed with PBS three times. T24 cells were fixed and developed using Prussian blue staining to visualize the presence of iron-oxide nanoparticles; fluorescent images showing FITC, the nuclear counterstain DAPI and merge respectively. FITC was visible by clusters of intense green fluorescence. Scale bar, 50 μm. **B**. The optical microscope images show 400× imaging of Prussian blue staining and nucleus fast red-counterstained and a magnified view of the black outlines the area. The majority of the blue granules can be seen inside the cytoplasm of the cell. Scale bar, 50 μm. **C**. TEM images of T24 cells and SV-HUC cells incubated with SPIO and SPIO-R11. A strong uptake of SPIO-R11 was observed and the quantity in T24 cells was much more compared to that in SV-HUC cells, whereas there was no significant uptake for SPIO particles. SPIO-R11 can be seen in vesicles and lysosome of cells. Magnification was at 100,000×.

In order to further verify SPIO-R11 cellular uptake and investigate intracellular localization, we observed the FITC staining of T24 cells after Prussian blue staining without nuclear fast red. As showed in Figure 3A, the intracellular localization of FITC as measured by fluorescence microscopy show that there is strong FITC staining in T24 cells after incubated with SPIO-R11 at 50 μg/mL of Fe concentration. The FITC staining is distributed in clusters throughout the cytoplasm and nuclei of the cells, among which the distribution of cytoplasm seem predominate. The cells were counterstained with nuclear fast red and observed by light microscopy (Figure 3B). Careful inspection shows that the majority of the intracellular blue color is accumulated around the perinuclear region. TEM gave insight into the subcellular localization of the particles in cells after incubation with SPIO and SPIO-R11. Figure 3C clearly shows that a large amount of the electron dense iron cores of the SPIO-R11 were internalised in vesicles and lysosome of the T24 cells. In line with the histologic findings, the quantity of SPIO-R11 cellular uptake by SV-HUC cells is much less compared to that by T24 cells and no significant uptake for SPIO is observed in both the cell lines. In addition, no evidence of cytotoxicity is observed in both the cells at the dosage used for incubation.

### Cytotoxicity study of SPIO and SPIO-R11

**Figure 4:**
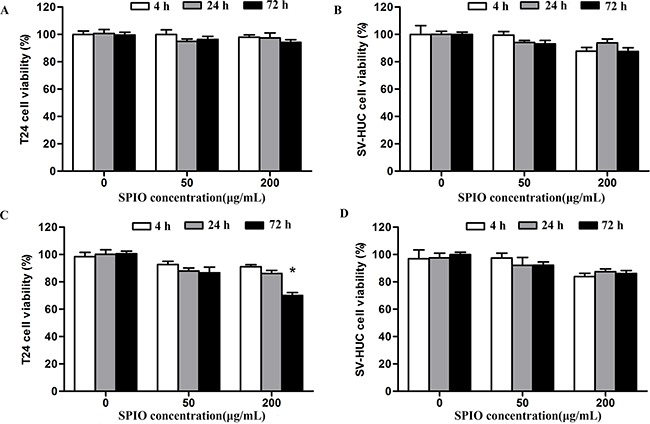
Cytotoxicity test of SPIO and SPIO-R11 **A**. The cytotoxicity on T24 cells and **B**. SV-HUC cells 4 h, 24 h and 72 h after SPIO incubation periods compared with controls at different Fe concentrations. **C**. The cytotoxicity on T24 cells and (B) SV-HUC cells 4 h, 24 h and 72 h after SPIO-R11 incubation periods compared with controls at different Fe concentrations. The decrease of cell viability was observed only in T24 cells incubated with SPIO-R11 at the highest Fe concentrations of 200 μg/mL after 72 h incubation periods. *Columns*, mean; *bars*, SD. *, *P* < 0.05.

The cytotoxicity of nanoparticles before and after surface modification with R11 was evaluated by MTT assay on T24 cells and SV-HUC cells. Figure 4A and 4B show that the SPIO were not toxic to the tested concentrations for SV-HUC and T24 cells. Even after 72 h of incubation at 200 μg/mL of Fe concentration, there was 94.2±4.0% and 87.5±5.4% viability of T24 and SV-HUC cells respectively, which did not differ from the percentage of viable control cells. Further, the SPIO-R11 showed no significant cytotoxicity on SV-HUC cells at the tested concentrations even at the longest incubation period of 72 h (*P* > 0.05). However, the SPIO-R11 showed decrease (*P* < 0.05) in the viability of the T24 cells at the highest Fe concentration of 200 μg/mL after incubation for 72 h compared with the controls.

### *In vitro* MRI studies

**Figure 5:**
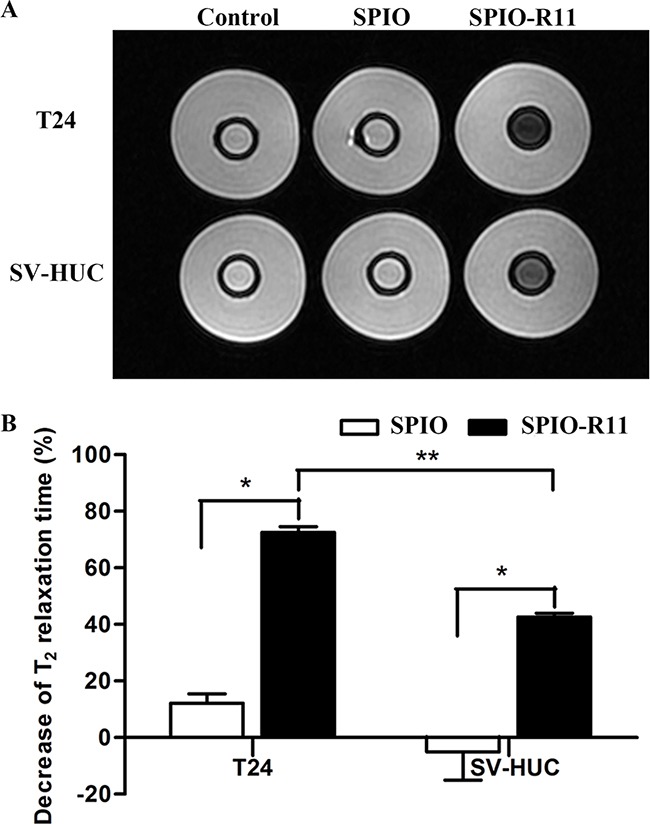
A. T_2_-weighted MR image of cells in gelatin (5×10^6^ cells/mL) before and after 4 h incubation with SPIO and SPIO-R11 (50 μg/mL of Fe concentration) Compared with the sample containing cells that were incubated with SPIO, the SI is clearly lower when cells were incubated with SPIO-R11. This decrease of SI was more obvious in T24 cells compared to SV-HUC cells. **B**. Significant decrease in MR T_2_ relaxation times with SPIO-R11 compared to SPIO. Moreover, after incubation with SPIO-R11, T24 cells showed a more pronounced decrease in MR T_2_ relaxation time than SV-HUC cells. *Columns*, mean; *bars*, SD. *, *P* < 0.05; **, *P* < 0.001.

To further verify the ability to enter bladder cancer cells using SPIO-R11 and whether this ability is sufficient to generate detectable contrast on *T_2_*-weighted MR, we performed preliminary *in vitro* cell imaging using a 3.0T MR scanner. Representative *T_2_*-weighted images before and after nanoparticle incubation are shown in Figure 5A. Both T24 and SV-HUC cells incubated with SPIO-R11 gave a darker image than control cells and SPIO-incubated cells, this enhancement of darkness in *T_2_*-weighted MR image is more obviously in T24 cells compared to that in SV-HUC cells. Then the decrease of *T_2_* relaxation times of the cells incubated with nanoparticles were measured in ROIs. Before incubation of the contrast agents, both the cells appeared with characteristically long *T_2_* relaxation times (123 ± 6 ms in T24 cells and 120 ± 4 ms in SV-HUC cells). As shown in Figure 5B, after 4 h incubation, the decrease of *T_2_* relaxation time was 12±3% for T24 incubated with SPIO and significantly lower for the T24 cells that were incubated with SPIO-R11 (73±2%). A similar results was obtained in SV-HUC cells after incubation with SPIO or SPIO-R11 (the decrease was -5%±10 and 42%±1 respectively). Though the *T_2_* relaxation times of T24 cells before and after incubation with SPIO are statistically different (the decrease was 12±3%, *P* < 0.05), it is not visually obvious Figure 5B. Additionally, after incubation with SPIO-R11, T24 cells showed a more pronounced decrease in *T_2_* relaxation time than SV-HUC cells (*P* < 0.001).

## DISCUSSION

At least 50% of patients with a history of bladder cancer have recurrences, so rigorous surveillance is necessary [3]. The application of MRI for efficiently monitoring small tumors has been hampered because of the low sensitivity of MRI contrast agents. Conventional small molecular *T_1_* gadolinium based contrast agents or chemical exchange saturation transfer (CEST) agents, such as Gd-DTPA or Eu-DOTA-4AmCE, however, have limited sensitivity of detection (>μmol/L), which makes it a considerable challenge to image tumor at much lower concentrations (e.g., *in vivo* targeting of tumor markers at a low physiologic concentration of nmol/L)[38, 39]. SPIO have shown significantly improved sensitivity because of their strong perturbation to the local magnetic field. Thus in this study, R11-conjugated, cross-linked dextran-coated SPIO were developed and their high uptake by bladder cancer cells was investigated *in vitro*. In contrast to previous molecular MRI approaches using nanoparticles with various coating layers, such as polymers [40, 41], dendrimers [42], and polysaccharides [43], the SPIO used in this study were coated with cross-linked aminated dextran. This coating provides the significant advantages that the presence of amino groups on the particle surface can be used to covalently attach specific ligands easily and that dextran is clearly biocompatible and biodegradable, thus providing basis for direct clinical applicability. The FITC-tagged R11 peptides were conjugated to the amino group of a dextran-coated SPIO using SPDP, one of the most popular heterobifunctional crosslinking agents.

The characterization of nanoparticle indicated that the R11 modification process had no effect on the core size, morphology and distributed of nanoparticle. Although the hydrodynamic size of the nanoparticles were increased by 13 nm after surface modification with R11, they are in the range of the ideal size [44]. The PDI of the nanoparticles suggested that the SPIO-R11 were still well-dispersed and stable in the solution compared to SPIO. The zeta potential values provide information about nanoparticle colloidal stability. Nanoparticles with a surface charge greater than +20 mV or less than -20 mV have a good stability, while nanoparticles with a lower surface charge easily aggregate due to Van Der Waal interparticle attractions [45]. In this study, the zeta potential measurements showed that nanoparticles modified with R11 have a high negative charge on the surface, which helps the nanoparticles stability by increasing interparticle repulsive forces [46]. The *r_2_* value is a standardized contrast-enhancement indicator at 3.0 T, the SPIO-R11 were found to have a significantly decreased *r_2_* value compared to SPIO. It can be explained by the quantum-mechanical outer-sphere theory. For a certain sized SPIO core, larger hydrodynamic size indicates larger core/shell ratio and lower *T_2_* relaxivity as suggested by Tong *et al*. for polymer coating [47]. Nevertheless, the *r_2_* value of SPIO-R11 is still much higher than those of commercial SPIO [12].

Following characterization, *in vitro* studies were conducted to test the extent of cellular uptake of the nanoparticles. Because incubation time varied in previous studies [32, 33, 37, 48], we initially used the Prussian blue iron staining method (Perls acid ferrocyanide) to detect iron within cells incubated with SPIO-R11 to determine the optimal time. The Prussian blue staining reduces ferric iron to the ferrous state by forming a blue precipitate [49]. The results clearly indicated that the highest difference in the uptake of SPIO-R11 was found at 4 h time points. After 4 h incubation, the difference in uptake of SPIO-R11 was less pronounced. A key consideration for the use of SPIO-R11 as an MRI contrast agent in targeted bladder cancer imaging is specific and high cellular uptake by bladder cancer cells. From the *in vitro* evaluation of the cellular uptake of two nanoparticles (SPIO-R11 and SPIO) by Prussian blue staining, we showed that there was a strong intracellular uptake for SPIO-R11 and the quantity in T24 cells was much more compared to that in SV-HUC cells, whereas there was no significant uptake for SPIO particles. This phenomenon was also confirmed by TEM and MR imaging *in vitro*, proving that surface modification with R11 facilitates high and specific uptake for SPIO in bladder cancer cells. The intracellular localization of SPIO-R11 as measured by fluorescence microscopy showed cytoplasmic and nuclear distribution, while Prussian blue staining and TEM showed cytoplasmic distribution with complete exclusion from the nucleus. We assume that this difference in the intracellular distribution of the FITC staining and the SPIO core particle may be due to the cleavage of disulfide linkage between peptide and nanoparticle. According to previous reports, the disulfide linkage is a reducing-environment-sensitive, reversible chemical bond and it is possible that cleavage of this bond occurs under the reducing conditions of the cytoplasm following internalization, and then the peptide is released from the particle and trapped by nuclear [50, 51]. Thus, the improvement of the stability of linkage between peptide and nanoparticle is one important aim of our ongoing research. Nevertheless, such cleavage does not affect our central conclusion: the uptake of the SPIO by bladder cancer cells can be greatly enhanced by the attachment of R11 peptides to that nanoparticles. Moreover, TEM analysis clearly showed that SPIO-R11 were distributed in vesicles and lysosome in the cytoplasm rather than localized in the nucleus, mitochondria, endoplasmic reticulum, Golgi apparatus, or any other cellular organelle, which could protect cells from damage and indicated that the SPIO-R11 are internalized into the cells possibly through endocytosis uptake [52, 53], though not investigated here.

Cytotoxicity of intracellular carriers is one of the most important parameters to be evaluated. Using MTT assay the SPIO-R11 appeared safe with SV-HUC cells at all concentrations even up to 72 h though some decrease in cell viability was observed with T24 cells at 200 μg/mL concentration. The decrease in T24 cells viability is likely due to internalisation of an enormous amount of nanoparticles into cells, which leads to an increased concentration of cellular iron and generates potentially toxic reactive oxygen species (ROS) through the Fenton and Haber-Weiss reactions [54]. These observations are in accordance with previous studies where SPIO exhibited low level of toxicity and biocompatibility up to 1 mg/mL concentration [48, 55, 56]. Additionally, our previous study demonstrated that R11 peptides were specific and efficient in human bladder cell lines and bladder tumor model by intraperitoneally injection with no significantly cytotoxic effect on normal bladder cells or other normal tissue, indicating the excellent cytocompatibility of R11 peptides as well [57]. Both the current results and previously reports demonstrate that R11 peptides as well as SPIO-R11 are safe and could potentially be used for further *in vivo* studies.

SPIO contrast agents can enhance MR contrast by shortening water proton *T_2_* relaxation times, leading to hypointense signals at locations where the nanoparticles accumulate. Therefore, we mainly consider the *T_2_* shortening induced by the SPIO nanoparticles. Through optimization studies, we have adopted an incubation procedure that uses incubation of cells with 50 μg/mL of nanoparticles for 4 h followed by extensive washing. Although this protocol does not necessarily result in the highest intracellular uptake possible, it represents a compromise of uptake efficacy, timeliness and cytocompatibility. The measurement of *T_2_* relaxation times of cells in gelatin by MR system proved the much larger enhancement of SPIO-R11 compared with the SPIO. Indeed, after SPIO-R11 incubation, there was a more pronounced decrease in *T_2_* relaxation times in T24 cells than in SV-HUC cells. Because of enhanced permeability and retention (EPR) effect of tumor vessels, the blood concentration of nanoparticles at the tumor site is higher than that in normal tissue, which may result in greater contrast effect [58]. In addition, although there was a 12% decrease in *T_2_* relaxation times in T24 cells after SPIO incubation (*P* < 0.05), the difference of *T_2_*-weighted MR images was not enough to clearly observe. This decrease can be explained by the fact that tumor cells have a stronger capability of endocytosis compared to normal cells, but the amount of nanoparticles internalised in the cell in this way was much smaller compared to R11-mediated cellular uptake. Our results, taken together with the MR relaxation data, Prussian blue staining, TEM data showing high uptake of SPIO-R11 by bladder cancer cells, suggest that R11-mediated cellar uptake may be a useful strategy for the targeted diagnosis of bladder cancer. These experiments established an *in vitro* framework for our subsequent *in vivo* studies aiming to detection of bladder cancer by MRI.

In conclusion, we successfully developed an R11-modified SPIO, which has good physicochemical properties and biocompatibility. Results from Prussian blue staining and TEM revealed that SPIO-R11 were efficiently taken up by bladder cancer cells and distributed in vesicles and lysosome in the cytoplasm. Moreover, *in vitro* MRI studies showed that SPIO-R11 significantly increased MR imaging contrast in T24 tumor cells over the SPIO. Therefore, we believe that SPIO-R11 have excellent promise to be a bladder cancer-specific MR imaging agent. Nevertheless, some issues such as pharmacokinetic properties and MR imaging *in vivo* using SPIO-R11 need to be further investigated in our next work.

## MATERIALS AND METHODS

### Synthesis of SPIO-R11

Aminated SPIO was purchased from BioPAL (Bio Physics Assay Laboratory Inc, MA, USA). R11 (FITC-Ahx-G-*RRRRRRRRRRR*-GGG-C-NH_2_) peptide was synthesized by automated peptide synthesizer using standard solid-phase chemistry and modified by treatment with FITC. The italicized amino acid residues are the membrane translocation sequence. R11 peptide was purified to >95% purity by reverse-phase high-performance liquid chromatography. The structure of synthesized peptide was confirmed by mass spectrometry.

Peptide-nanoparticle complex was prepared as previously described [15, 32, 37]. To 1 mL of amino-SPIO (5 mg Fe, 89 μmmol) was added 0.5 mL of 0.1 M phosphate buffer (pH=7.4), and 0.25 mL of N-succinimidyl-3-(2-pyridyldithio) propionate (SPDP) (Heowns Biochen Technologies, Alpharetta, GA, USA) dissolved in DMSO (20 mM, 5 μmol). The mixture was allowed to stand for 2 h at room temperature. Low molecular weight impurities were removed by a Sephadex G-25 column (20×1.5 cm; Sigma-Aldrich Co., St. Louis, MO, USA) and the void volume with iron oxide was collected. The process can be monitored by atomic absorption spectrophotometer. 1.28 mg of peptide dissolved in 1 ml deionized water was added to the iron oxide and the mixture was allowed to react for 4 h at room temperature. The solution was applied to Sephadex G-25 columns as above and the excluded volume containing SPIO-R11 saved.

### Characterization of nanoparticles

To assess the size and morphology of nanoparticles, the R11-unmodified SPIO and R11-modified SPIO were characterized using a H-7650 transmission electron microscope (TEM; Hitachi Tokyo, Japan) operating at 80 kV. The samples were dropped onto carbon-coated copper grids, and air-dried before TEM measurements. To further determine the hydrodynamic diameter, polydispersity index (PDI) and surface charge of the nanoparticles, SPIO and SPIO-R11 were characterized using Malvern Zetasizer Nano-ZS device. Transverse relaxivity measurement was conducted on a Varian 3T MRI system (Signa HDxt, General Electric Medical System, Milwaukee, WI, USA) using a circularly polarized quadrature knee coil (Clinical MR Solutions, Brookfield, Wis). The transverse relaxation time *T_2_* of the nanoparticles were measured in 1% agarose with concentration from 0.06 to 0.3 mM [Fe], using *T_2_* Mapping sequence: at TR 1500 ms and TE (76, 66.5, 57, 47.5, 38, 28.5, 19 ms). Slice thickness was 2 mm. The field of view (FOV) was 100 mm. Then *T_2_* maps were generated on AW 4.4 workstation (GE Healthcare). For each sample, three *T_2_* measurements were performed. The *r_2_* values were calculated based on 1/*T_2_* versus iron concentration.

### Cell culture

The human bladder cancer cell line T24 and the immortalized bladder epithelial cell line SV-HUC obtained from American Type Culture Collection (ATCC, Manassas, VA) were used in this study. The T24 and SV-HUC cells were maintained in Dulbecco's modified Eagle's medium (DMEM) (Invitrogen, Carlsbad, CA) supplemented with 10% fetal bovine serum in a humidified incubator at 37°C with 5% CO_2_. The cells were regularly monitored with an inverted light microscope, and the culture medium was changed every 2 d until they reached at least 90% confluence.

### Labeling of bladder cells with nanoparticles and prussian blue staining

To determine the optimal time of incubation, the T24 cells were counted using a regular hemocytometer and seeded at a density of 5×10^4^ cells per well in a 24-well plate. The T24 cells were incubated with 0.5 mL culture medium containing SPIO-R11 or SPIO at an iron concentration of 50 μg/mL for different time intervals (2 h, 4 h, 6 h, 8 h and 12 h). For Prussian blue staining, the labeled T24 cells were fixed with 4% paraformaldehyde (Sigma-Aldrich Co.), washed in PBS, and then incubated with a mixture (50:50, v/v) of 2% potassium ferrocyanide and 2% hydrochloric acid (Perls reagent) for 30 min. Counterstaining was performed by incubating the cells with nuclear fast red for 3 min after washing with PBS.

To evaluate the R11-mediated cellular uptake efficiency of SPIO, the T24 and SV-HUC cells seeded in 24-well plates were incubated with various concentrations of the SPIO-R11 (0 μg/mL, 25 μg/mL, 50 μg/mL, 100 μg/mL and 200 μg/mL). The incubation time was determined by the above results. SPIO-labeled T24 and SV-HUC cells served as controls. After incubation, Prussian blue staining was performed as above. The prepared samples were observed under an inverted fluorescence microscope (Eclipse TE 2000-U, Nikon, Kyoto, Japan) equipped with a high-resolution CCD camera (CV-S3200, JAI Co., Japan).

### Subcellular localization of the nanoparticles

Labeling of cells with iron nanoparticles was done by 4 h (from previous time study) incubation with 50 μg/mL of nanoparticles in cell culture medium. The cells were washed with PBS three times to remove any free particles before use. Prussian blue staining without nuclear fast red was done on fixed cells (4% paraformaldehyde) after labeling. The cells were counterstained with DAPI dye (100 nM; Sigma-Aldrich Co.) to reveal their nuclei. Fluorescence microscopy (Eclipse TE 2000-U, Nikon, Kyoto, Japan) was used to confirm that SPIO-R11 labeling was truly intracellular. Then the cells were counterstaining with nuclear fast red and observed at ×10 magnification (Olympus BX40, Japan). For TEM, cells were fixed with glutaraldehyde in 0.1 M sodium cacodylate (pH 7.2) at 4°C overnight. They were then stained with 1% osmium tetroxide, dehydrated in a graded ethanol-water series, embedded in epoxy resin and processed for ultrathin sectioning. Some of ultrathin sections stained with uranyl acetate and lead citrate. Micrographs were taken with a Hitachi 7650 transmission electron microscope at 80 kV. The magnification indicator was routinely controlled by the use of a grating replica.

### Cytotoxicity assay

The 3-(4,5)-dimethylth-iahiazo(−z-y1)-3,5-dipheny tetrazoliumromide (MTT) assay evaluates cell proliferation to test cytotoxicity. The T24 and SV-HUC cells were seeded into 96-well plates at 2×10^4^ cells per well and 4×10^4^ cells per well respectively. After incubation for 24 h, the cells were washed with PBS and co-incubated with SPIO-R11 or SPIO at different Fe concentrations (50-200 μg/mL). After 4 h, 24 h and 72 h, the culture medium was removed, and 200 mL of the MTT solution (final concentration: 0.5 mg/mL; Sigma-Aldrich Co.) was added. The cells were then incubated for 4 h, and 150 μL of DMSO was added. The plates were incubated for another 15 min. Finally, absorbance was measured at 490 nm on a microplate spectrophotometer (Bio Tek Instrument Inc., USA). Each result is an average of data from five wells and the nanoparticles-untreated cells served as controls.

### MR imaging of cells in gelatin

Phantoms were constructed for MR imaging that consisted of 5×10^6^ cells embedded in agar to prevent drying and susceptibility artifacts [32]. The T24 and SV-HUC cells were incubated with SPIO-R11 or SPIO and sedimented cell pellets containing 5×10^6^ cells were resuspended in 1 mL of 1% low-melting warm agarose and placed into holes previously stenciled into the agarose. Each well was then sealed with additional agarose. Unlabeled cells (5×10^6^ cells/pellet) served as controls. *T_2_*-weighted spin-echo (SE) images of cells were obtained using a Varian 3T MRI system (Signa HDxt, General Electric Medical System, Milwaukee, WI, USA) using a circularly polarized quadrature knee coil (Clinical MR Solutions, Brookfield, WI) under the following conditions: time of repetition/time of echo, 4000/60 ms; flip angle, 90°; field of view, 100 mm; echo train length, 12; section thickness, 2.5 mm. Signal intensity was measured in defined regions of interest (ROIs) in the cells. The decrease of *T_2_* relaxation times were calculated using *T_2_* relaxation times measurements of nanoparticles-labeled (*T_2_* lab) and -unlabeled cells (*T_2_* unlab) according to the following formula: Decrease of T_2_ relaxation times (%) = [(*T_2_* unlab- *T_2_* lab) / *T_2_* unlab]×100.

### Statistics

Statistical analysis was done using GraphPad Prism 5 (GraphPad Software). Statistical comparisons were assessed using Student's *t* test. Results were considered to be statistically significant at *P*< 0.05.

## SUPPLEMENTARY FIGURES


