# Supplementary material for: Synthesis of a cell penetrating peptide modified superparamagnetic iron oxide and MRI detection of bladder cancer
Source: Oncotarget. 2016 Nov 24;8(3):4718–29. doi: 10.18632/oncotarget.13578 (PMC5354866; doi:10.18632/oncotarget.13578)
Supplement: Supplementary file 1 [file oncotarget-08-4718-s001.pdf]

Synthesis of a cell penetrating peptide modified superparamagnetic iron oxide and MRI detection of bladder cancer

SUPPLEMENTARY FIGURES

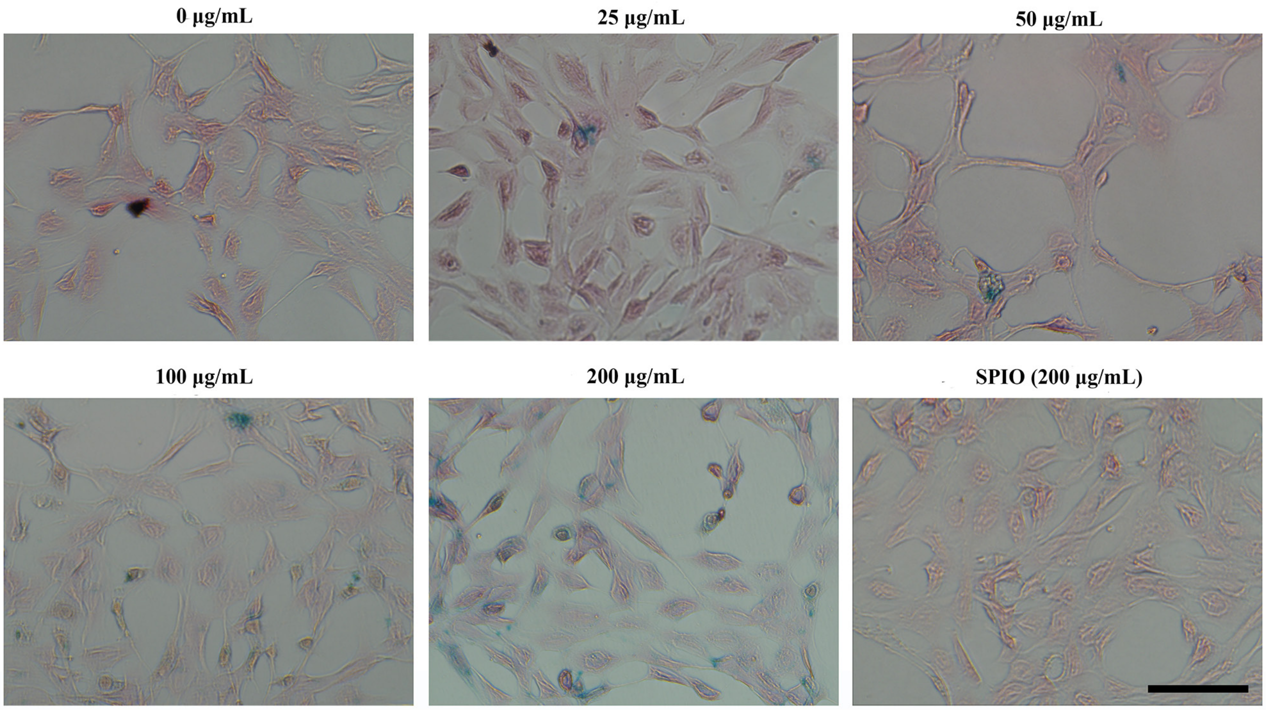

Supplementary Figure S1: Prussian blue-stained and nucleus fast red-counterstained SV-HUC cells incubated with various iron concentrations of SPIO-R11 (0-200 µg/mL) and SPIO (200 µg/mL). The bar in the bottom right corner represents 50µm.

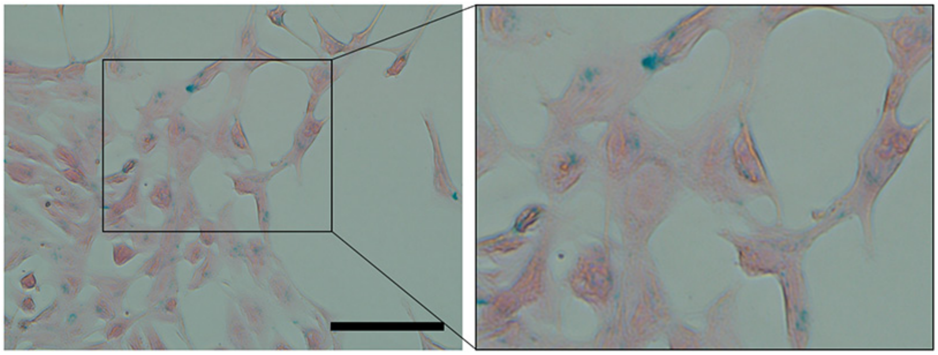

Supplementary Figure S2: The optical microscope images of SV-HUC cells incubated with SPIO-R11. The magnified view of the black outlines the area shows that the blue granules localize within the cytoplasm and are not found in the nucleus. Scale bar, 50 µm.
